# Enhancing sweet potato production: a comprehensive analysis of the role of auxins and cytokinins in micropropagation

**DOI:** 10.1007/s00425-025-04650-z

**Published:** 2025-03-04

**Authors:** Gideon Adu Donyina, Adrienn Szarvas, Vincent Agyemang Opoku, Edit Miko, Melinda Tar, Szilárd Czóbel, Tamás Monostori

**Affiliations:** 1 Doctoral School of Environmental Sciences, University of Szeged, Dugonics Square 13, Szeged, 6720 Hungary; 2 Institute of Plant Sciences and Environmental Protection, University of Szeged, Andrássy Út 15, Hódmezővásárhely, 6800 Hungary; 3 Institute of Animal Sciences and Wildlife Management, University of Szeged, Andrássy Út 15, Hódmezővásárhely, 6800 Hungary; 4 Department of Crop Science, School of Agriculture, College of Agriculture and Natural Sciences, University of Cape Coast, Cape Coast, Ghana; 5 Department of Agroecology, Faculty of Technical Sciences, Aarhus University, Tjele, Denmark

**Keywords:** *Ipomoea batatas*, Micropropagation, Plant growth regulators, Tissue culture

## Abstract

**Main conclusion:**

This review emphasizes the prevalent auxins and cytokinins used in sweet potato micropropagation, their optimal concentrations for effective in vitro regeneration, various propagation techniques, and Africa's potential to improve sweet potato production.

**Abstract:**

*Ipomoea batatas* (L.) Lam., or sweet potato, is a robust, nutritious, and adaptable crop traditionally propagated through conventional methods. These techniques, however, have limitations, prompting the adoption of micropropagation as an efficient alternative for producing healthy, cost-effective plantlets in reduced time. This review critically evaluates the influence of auxins and cytokinins, the most frequently utilized plant growth regulators (PGRs), in enhancing sweet potato micropropagation protocols. The study examines the crop's origins, distribution, and cultivation practices, as well as the morphophysiological effects of PGRs on sweet potatoes. Our analysis reveals that 6-benzylaminopurine (BAP) and N6-benzyladenine (BA) are the predominant cytokinins, while naphthaleneacetic acid (NAA) and indole-3-butyric acid (IBA) are the primary auxins employed in sweet potato micropropagation. The review also proposes strategies for increasing production, particularly in Africa, and identifies areas requiring further investigation to better understand how these growth regulators impact the physiological development and response of sweet potatoes to environmental stress. This comprehensive assessment contributes to the expanding knowledge base on sweet potato micropropagation and offers valuable insights for researchers and practitioners in the field.

## Introduction

**Fig. 1:**
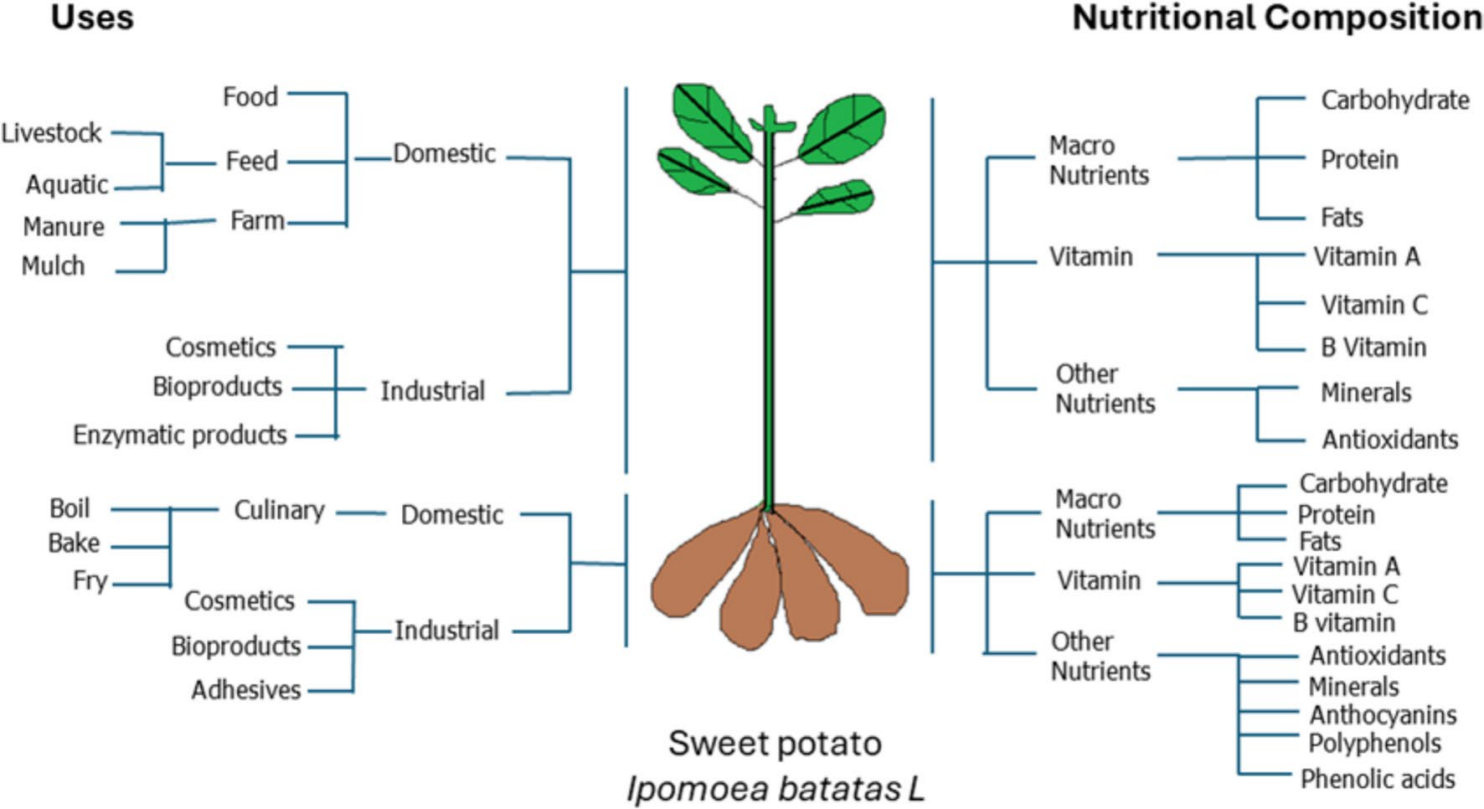
Summary of domestic and industrial uses of sweet potato vines and storage roots (left). Nutritional composition of sweet potato vines and storage roots with emphasis on its macronutrients, vitamins, and other nutrients (right)

*Ipomoea batatas* (L.) Lam, commonly known as sweet potato, is valued for its socioeconomic status, nutritional value, and ability to adapt to various agroecological conditions. This aforementioned relevance makes the crop unique to other species within the Ipomoea genus (Monostori and Szarvas 2015; Drapal et al. 2019; Behera et al. 2022; Leite et al. 2022; Tadda et al. 2022). Sweet potato is ranked sixth among the major staple crops worldwide (Monostori and Szarvas 2015; Drapal et al. 2019). Sweet potatoes remain an excellent source of essential dietary nutrients and are valued for their health benefits (Fig. 1). Research has shown that consuming sweet potato tubers, particularly those with orange or purple flesh, can effectively combat inflammation, obesity, and cancer. This is because of their rich content of vital nutrients, including carotenoids, polyphenols, anthocyanins, and phenolic acids (Dolinski, 2013; Leite et al. 2022; Pérez-Pazos et al. 2023). Furthermore, the plant's vines and foliage are recognized as rich sources of vitamins, minerals, and various bioactive substances, making them a notable feed option for livestock, especially swine. (Islam 2006; Mohanraj and Sivasankar 2014; Cartabiano-Leite et al. 2020; Tadda et al. 2022) (Fig. 1). All of these factors have contributed to their wide usability. One of the world's most crucial food crops for human consumption is the sweet potato. It is predominantly cultivated in developing nations with low per-capita income levels. Consequently, boosting sweet potato production is viewed as a strategy for enhancing food security and alleviating poverty among the less affluent segments of both rural and urban populations (Afzal et al. 2021). Thus, enhancing sweet potato production is of paramount relevance in achieving sustainable development goals (SDGs 1 and 2) aimed at zero hunger and improved livelihoods (Asante et al. 2024; Opoku et al. 2024).

Sweet potatoes are believed to have originated from the tropical regions of Americas (Mohanraj and Sivasankar 2014; Monostori and Szarvas 2015; Shen et al. 2023). However, it was later introduced in several Asian countries including China, India, Japan, and Vietnam. China is currently the global leader in sweet potato production (Cartabiano-Leite et al. 2020; Leite et al. 2022). History records that the crop was introduced into the European continent by the famous Italian adventurer Christopher Columbus through his exploration activity in the 1490 s (Cartabiano-Leite et al. 2020) and later to the African continent in the sixteenth century during Columbian exchange. Sweet potato has gained interest since its introduction in Africa, making it a crucial component of African crop production and utilization. While some prefer to fry, boil, or bake tuberous roots, others choose to process them into flour for the preparation of cakes, bread, chapatti, and biscuits (Vollmer et al. 2023).

Sweet potatoes are commonly cultivated asexually using stem cuttings (Dolinski 2013; Kirakosyan et al. 2013; Pérez-Pazos et al. 2023), and storage roots, which tend to produce another propagation material called slips (Behera et al. 2022). The use of these readily available planting materials tends to provide farmers with inexpensive propagation methods. However, the associated problems cannot be overemphasized. Slow propagation rates, high labor costs, and pathogen-infested propagules (Ogero et al. 2012; Tadda et al. 2022; Pérez-Pazos et al. 2023) are a few examples of the challenges of using the conventional propagation method (Behera et al. 2022). Moreover, this method hinders continuous production of uniform and healthy propagules for commercial purposes. Therefore, the use of in vitro micropropagation presents a reliable remedy, as it ensures the rapid propagation of improved and healthy propagules on a large scale with relatively less space and equipment.

In vitro micropropagation is a plant biotechnological method specifically related to plant cell and tissue culture, and is used to rapidly multiply planting materials for various species, including sweet potatoes. It offers a reliable means of producing healthy and genetically improved planting materials that can withstand environmental stress. This involves careful selection of a suitable explant and proper culturing on a growth medium with the help of sterile instruments under aseptic conditions. Additionally, thorough treatment of explants to render them pathogen-free is possible (Dolinski 2013; Behera et al. 2022; Hu et al. 2022; Pérez-Pazos et al. 2023; Vollmer et al. 2023).

In sweet potato micropropagation, various substances are used in the different explant disinfection protocols. These include sodium (or calcium) hypochlorite and 70% ethanol. Murashige and Skoog salts and vitamins form the most common culture medium (MS) used in sweet potato micropropagation in combination with sucrose (Murashige and Skoog, 1962). Other nutrient media used in several experiments included modified MS and Linsmaier–Skoog (LS) media. The culture medium may or may not contain plant growth regulators (PGRs), although several reports have highlighted the crucial role of these PGRs in the successful establishment of the culture (Behera et al. 2022). Auxins, cytokinins, gibberellins, abscisic acid, and ethylene form a common class of PGRs (Bari and Jones 2009). Auxins and cytokinins are the most commonly used PGRs in sweet potato micropropagation. Their synergistic combination with nutrient media enhances bud-breaking, shoot initiation, growth, root development, and overall plant regeneration. Several researchers have explored the individual or combined effects of plant growth regulators (PGRs) on sweet potato micropropagation. However, over- or under-application of these substances can result in undesirable plant regeneration characteristics. To aid the work of researchers and plant producers, this review focuses primarily on optimizing the use of auxins and cytokinins for efficient micropropagation of sweet potato. It summarizes the results of previous and current experimental studies on the micropropagation of sweet potatoes. Furthermore, it examines the impacts of auxins and cytokinins on physiological, morphological, and biochemical aspects, and identifies the optimal concentrations of these plant growth regulators for successful sweet potato micropropagation.

## Sweet potato production

**Fig. 2:**
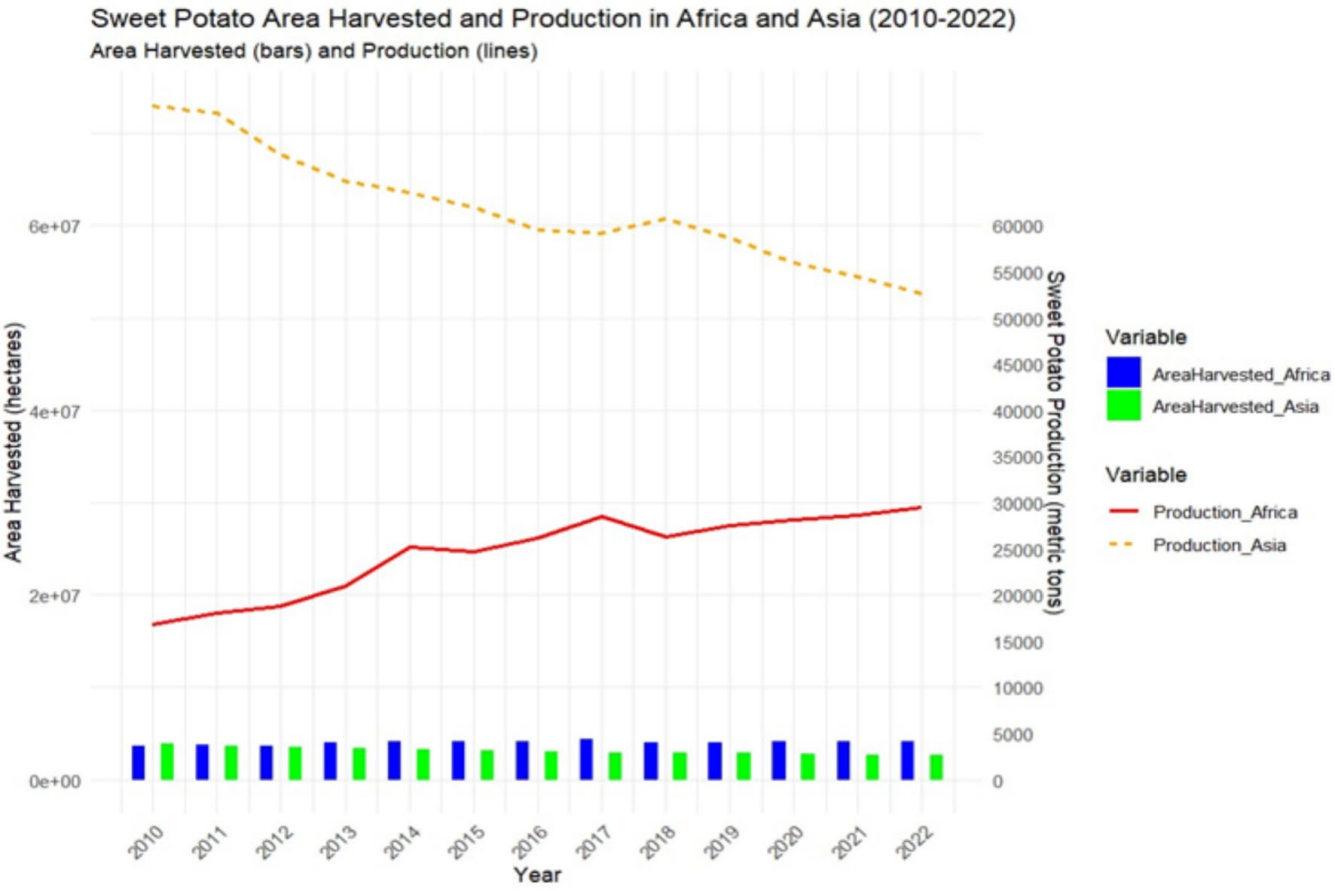
Trend of sweet potato production (2010 – 2022), indicating harvest and production areas in Africa and Asia. Source: FAO 2024)

Sweet potato [Ipomoea batatas (L.) Lam.], a resilient and economically significant crop, has garnered worldwide attention because of its high production levels. The majority of global sweet potato cultivation is concentrated in Asia and Africa, with these two continents accounting for 95% of the worldwide production. Among the continents producing sweet potatoes, Asia leads the way, followed by Africa, America, Oceania, and Europe, in descending order (Cartabiano-Leite et al. 2020). China has been consistently ranked as the largest producer of sweet potatoes, accounting for over 86% of global production, followed by some African countries such as Malawi, and Nigeria, with Malawi producing twice as much sweet potatoes as Nigeria (FAO 2024). Among the factors that contribute to the recognition of these countries as top producers of sweet potatoes are the availability of a wide array of genotypes, favorable ecological conditions, and appropriate cultivation or propagation methods. Due to the significant global interest in sweet potatoes, several breeders have successfully developed an array of varieties that suit local conditions and demands. For example, Opele, Apomuden, Kadyaubwerere, and Wagabolige are popular sweet potato genotypes in Africa. Although Asia has the largest share of sweet potato production, statistical records obtained from the Food and Agricultural Organization of the United Nations (FAO 2024) show that Africa has a larger harvested sweet potato area than Asia (Fig. 2). Therefore, the low yield can be attributed to inefficient cultivation techniques in Africa. Similarly, Ebem et al. (2021) reported that the low yield of sweet potatoes in Nigeria could be attributed to inefficient input and improper cultivation methods. This suggests that Africa has the potential to overtake Asia in sweet potato production, and with appropriate measures put in place to improve yield per unit area, this dream could become a reality. Agricultural policies that seek to leverage scientific findings in the provision of efficient production inputs, large-scale mechanization, and borderless trade in the African region can contribute significantly to the realization of this dream.

## Ecological requirements

As a tropical and subtropical crop (Dolinski 2013; Hu et al. 2022), sweet potatoes require a warm climate, with temperatures between 21 °C and 29 °C, and adequate sunlight exposure for optimal growth. Soils rich in organic matter, well-drained, and with a pH range of 5.0 to 6.5 are ideal for sweet potato cultivation because they promote good root development, tuber formation, and overall plant health (Monostori and Szarvas 2015; Behera et al. 2022). Sweet potatoes have specific nutrient requirements. Potassium (K) is the most crucial nutrient because it greatly affects tuber development, yield, and quality. This is followed by nitrogen (N), calcium (Ca), and phosphorus (P), in decreasing order of importance for sweet potato cultivation. Although nitrogen can significantly increase tuber yield, excessive application can result in uncontrolled foliage development at the expense of tuber development and may also cause cracking in tubers. Although required in lower amounts, calcium plays a functional role in enhancing the shelf life of sweet potato tubers (Bagdi et al. 2023). Therefore, it is recommended that soil testing be conducted for informed decisions regarding fertilizer application. Consistent moisture throughout the growing season, especially during tuber formation, is critical for optimum yield. However, overwatering must be avoided because it can cause root rot in the tubers.

## Sweet potato propagation methods

The selection of appropriate sweet potato propagation techniques is essential for successful cultivation and optimal yield. Various methods are employed in sweet potato farming to preserve genetic integrity and manage diseases. Among local and small-scale farmers, vine cutting is the most widely adopted approach for sweet potato production (Dolinski, 2013; Belachew et al. 2020; Behera et al. 2022; Kirakosyan et al. 2023). This traditional propagation method is predominantly used in tropical and subtropical regions. Farmers typically choose vines or stems based on visual assessment, divide them into segments containing at least four nodes, and plant these cuttings in the soil. However, this method is slower, seasonal, and often results in higher labor costs and potentially pathogen-infected propagules (Behera et al. 2022; Tadda et al. 2022). In temperate climates, the primary planting material consists of sprouts (slips) that grow from storage roots incubated in nurseries (Monostori and Szarvas 2015). This approach shares similar drawbacks with the vine-cutting method. Consequently, the quest for improved propagation techniques has led to the adoption of in vitro micropropagation, which can be integrated with conventional methods.

Micropropagation has revolutionized sweet potato production by offering several advantages, including the rapid multiplication of disease-free planting materials, genetic uniformity, and the ability to propagate elite varieties efficiently and sustainably (Dobránszki and Teixeira Da Silva 2010; Belachew et al. 2020; Dewir et al. 2020; Tadda et al. 2022; Vollmer et al. 2023). This involves culturing plant cells or tissues in a controlled environment. Micropropagation can be conducted through meristematic culture, somatic embryogenesis, adventitious organogenesis, callus-mediated organogenesis, protoplasts, root hair culture, anther culture, and ovary culture. However, as nodal explants tend to express higher responsiveness to shoot proliferation than other explants, meristematic culture is the most commonly used technique in sweet potato micropropagation (Behera et al. 2022). This method has played a crucial role in enhancing the quality and quantity of sweet potatoes worldwide. Using micropropagation techniques, researchers have been able to produce disease-free planting materials, conserve germplasms, and enhance the sustainability of sweet potato cultivation (Alula et al. 2018). Various factors, including advancements in tissue culture technology, the development of optimized growth media, and the selection of superior genotypes for propagation, have influenced the evolution of sweet potato micropropagation. Researchers have focused on refining micropropagation protocols to improve the efficiency and success rate of plant regeneration from tissue culture (Ogero et al. 2012; Belachew et al. 2020; Dewir et al. 2020; Vollmer et al. 2023).

**Fig. 3:**
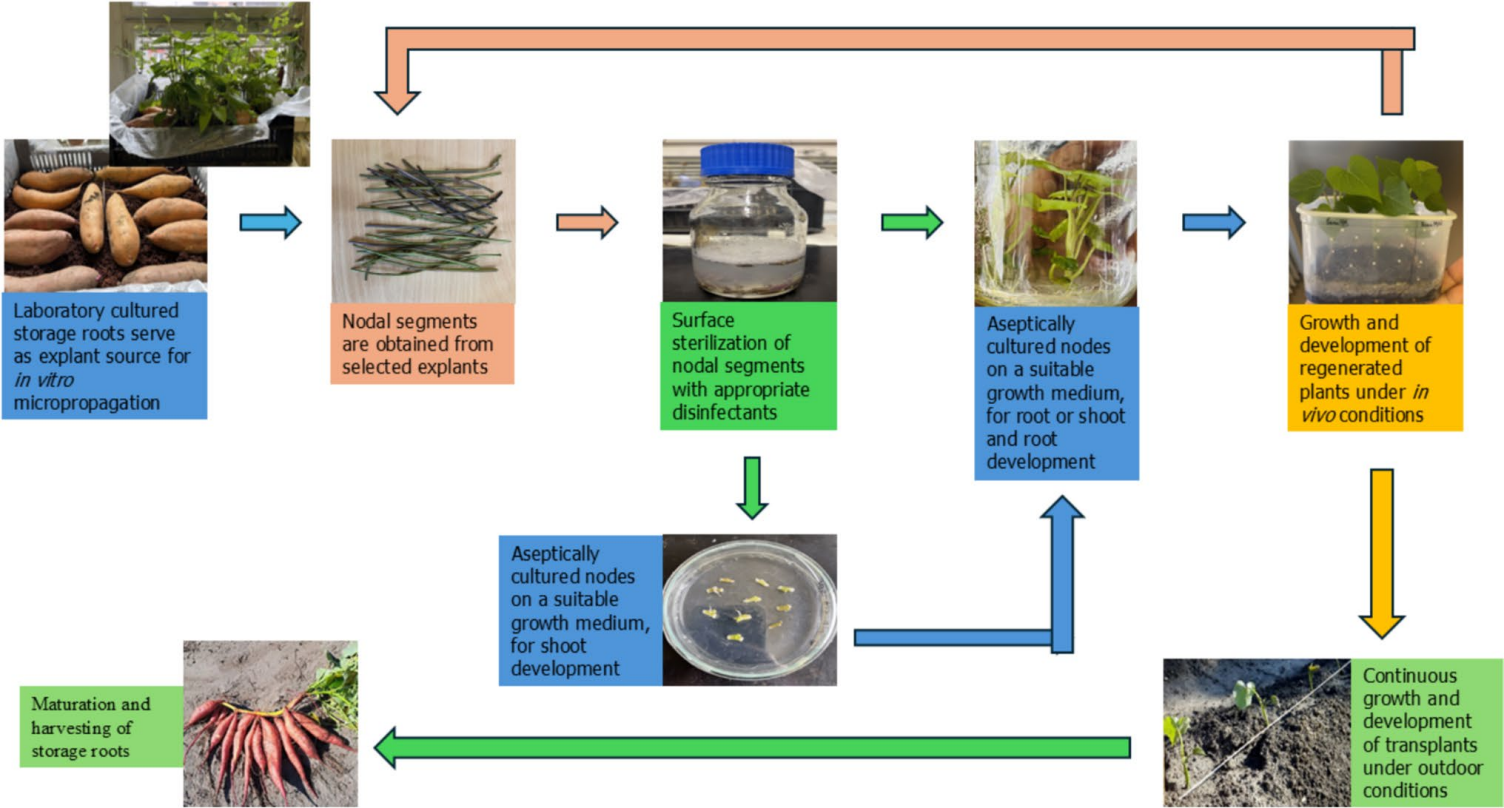
Schematic description of the stages involved in in vitro micropropagation of sweet potato using nodal segment

Several factors influence the success of the micropropagation. These include the selection of an appropriate explant, treatment mode of the explant to render it sterile, choice and composition of the nutrient medium, and overall aseptic conditions of the working environment (Fig. 3) (Chen et al. 2006; Doussoh et al. 2019; Pérez-Pazos et al. 2023). In addition to the commonly used MS medium and other frequently used media, such as the LS medium (Behera et al. 2022), some researchers, such as Ogero et al. (2012), have explored the use of various locally made nutrient media for micropropagation. Depending on the protocol used, plant growth regulators can be added to the nutrient medium.

## Plant growth regulators

Plant Growth Regulators (PGRs), also referred to as plant bioregulators, encompass a range of natural and synthetic substances capable of modifying plants' developmental or metabolic processes. These compounds are extensively utilized in agricultural practices to regulate plant growth and development (Ashok et al. 2019; Ahmed et al. 2022). PGRs can impact various developmental stages, including germination, flowering, fruit formation, ripening, abscission, and defoliation, as well as certain quality attributes like fruit size and weight, even when applied in minute quantities (Adil et al. 2018). Research has shown that PGRs play a crucial role in normal plant growth and development (Yang and Komatsu 2004). These substances have been observed to affect key indicators of plant health, influencing both anatomical structures and mitotic activity within plants (Zabolotnyi et al. 2021). Additionally, research has investigated the impact of micropropagation with plant growth regulators (PGRs) on sweet potato nutrition and performance. For example, Ghasemzadeh et al. (2016) observed substantial increases in total phenolic content (TPC), total flavonoid content (TFC), total anthocyanin content (TAC), total β-carotene content (TCC), and antioxidant activities in plants treated with PGRs. This effect may be due to PGRs' capacity to initiate crosstalk, thereby influencing the production of phytochemicals and secondary metabolites. Researchers have also utilized PGRs in micropropagation to enhance somatic embryogenesis and plant regeneration. The effects of PGRs on sweet potato somatic embryogenesis were evaluated by Sefasi et al. (2012), Manrique-Trujillo et al. (2013), and Masekesa et al. (2021). Likewise, Valery et al. (2014), Masekesa et al. (2016), and Arathi et al. (2019), among others, have examined how PGRs affect in vitro plant regeneration across various sweet potato genotypes. It is important to note that plant growth regulators are not considered nutrients or toxic substances (Bari and Jones 2009).

PGRs are not only essential for normal growth and development but also play a critical role in plant defense against pathogens and diseases. They are integral parts of plant signaling networks and are involved in regulating plant metabolism and growth under adverse environmental conditions, such as drought, cold, heat, and salinity stress (Kazan 2013; Overvoorde et al. 2010; Checker et al. 2018; Lee et al. 2019; Zhao and Li 2021). Plant Growth Regulators tend to significantly affect treated plants at various levels, including morphological, molecular, and physiological. External application significantly affects shoot elongation, plant responses to biotic and abiotic stresses, and endogenous hormone levels. They also interact with other signaling molecules to optimize plant development and metabolism, leading to metabolic reprogramming for the enhanced biosynthesis of secondary plant products.

## Auxins

The term "auxin," was derived from Greek and it means "to grow,". Although discovered in the late nineteenth century by Charles Darwin, it is reported to have been isolated in 1931 by Kögl and Haagen-Smit who also coined the term for a compound that they discovered had the potential to influence plant growth (Pacifici et al. 2015). Auxins are a group of plant growth regulators that influence various aspects of plant development, including cell elongation, apical dominance, root formation, and fruit growth (Rahman 2013; Gupta and Corpas 2021; Altaf et al. 2023). The most common auxin found in plants is indole-3-acetic acid (IAA) (Husen 2021), which is synthesized in the shoot apical meristem and young leaves (Aloni 2021).

During its operation as a regulator of plant growth development, auxin tends to induce the expression of three classes of genes: GH3 family, AUX/IAA family, and the small auxin-up RNA (SAUR) family. It has been found that the interaction between AUX/IAA proteins and transport inhibitor response 1 (T1R1) is crucial to the control of auxin signaling and response in plants. This was observed in the function of T1R1 as a facilitator of AUX/IAA repressor protein degradation, resulting in the activation of auxin-responsive genes (Saini et al. 2013; Lavy and Estelle 2016). The activation of these genes triggers certain physiological responses such as embryogenesis, cell elongation, and root initiation (Danilova et al. 2020).

## Role of auxins in sweet potato micropropagation

The utilization of auxins in sweet potato micropropagation has been a subject of investigation due to their critical role in promoting root initiation, elongation, lateral root formation, callus formation, and the overall growth of plant tissues. Auxins, particularly indole-3-acetic acid (IAA), have been examined for their effects on root development and differentiation in tissue culture. In sweet potatoes, auxins have been identified as essential for the tuberization process, influencing the formation of tubers, which are vital components of the plant storage organ (Mathura et al. 2023).

Research has demonstrated that the addition of synthetic growth regulators with auxin-like effects, such as α-naphthalene acetic acid (NAA), to the culture medium can enhance root initiation and overall growth of sweet potato plantlets. For instance, in the micropropagation of sweet potato genotypes such as 'Kullufo,' the inclusion of 0.5 mg/L of NAA in the medium resulted in successful (96.6%) root formation from regenerated shoots (Mengs et al. 2018). Through careful manipulation of the concentration and exposure of auxin in the culture medium, researchers can effectively promote the development of a well-established root system in micropropagated sweet potatoes. Auxin rapidly alters the transcript levels of numerous genes, suggesting that many of its effects are mediated by changes in gene expression. Elucidating the specific gene expression patterns and regulatory networks influenced by auxins in sweet potato tissue cultures can provide valuable insights into the mechanisms underlying root development and overall in vitro plant growth. The interaction between auxin and other growth regulators such as cytokinins is crucial for optimizing the micropropagation process in sweet potatoes. For instance, Kirakosyan et al. (2022) reported optimum sweet potato plant regeneration through a combination of auxin and cytokinin in their in vitro experiments.

## Cytokinins

**Fig. 4:**
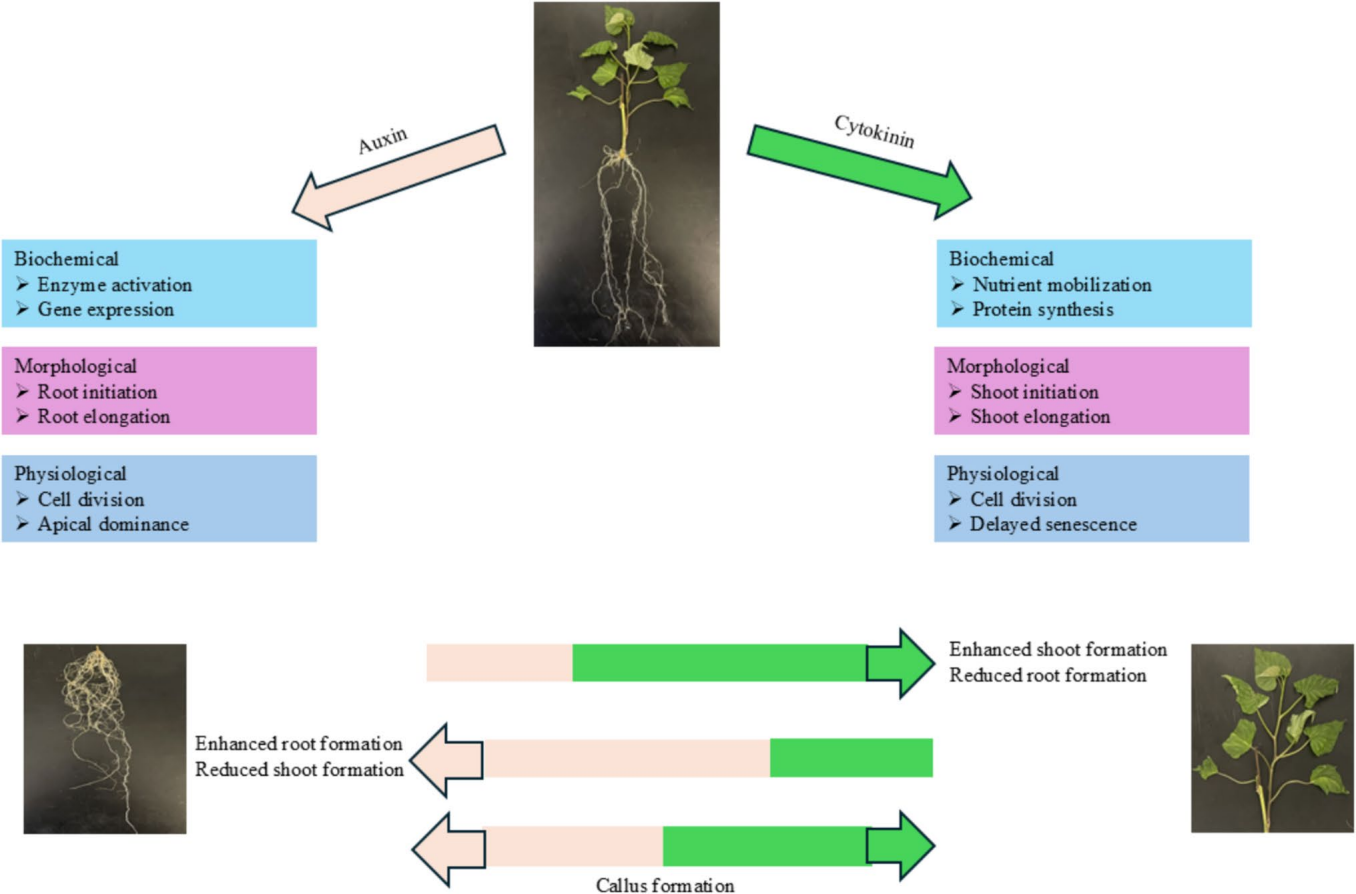
Morphological, physiological, and biochemical effects of auxin on sweet potato micropropagation (left panel). Morphological, physiological, and biochemical effects of cytokinins on sweet potato micropropagation (right panel). Their interactive effect under varied concentrations (bottom)

Cytokinins are adenine-derived signaling molecules involved in regulating processes, such as cell division, metabolism, chloroplast development, shoot and root development, leaf senescence, embryo and seed development, shoot initiation, vascular differentiation, and organogenesis (Müller and Sheen 2008; Magyar-Tábori et al. 2010; Kim et al. 2012; Gao et al. 2013; Chen et al. 2022) (Fig. 4). Isopentenyl diphosphate is a precursor for cytokinin synthesis. The biosynthetic process begins with the formation of isopentenyl diphosphate (IPP) via the methylerythritol phosphate (MEP) pathway in plastids. IPP was then converted into dimethylallyl diphosphate (DMAPP). DMAPP acts as a donor for the isopentenyl group. An enzyme called adenylate isopentenyl transferase (IPT) catalyzes the transfer of the isopentenyl group from DMAPP to the N^6^ position of adenosine monophosphate (AMP), producing an isopentenyl adenine (iP) ribonucleotide (Kurakawa et al. 2007; Khan, 2012; Spíchal 2012; Hoyerová Hošek 2020). Ribonucleotides are then dephosphorylated to release free bases such as trans-zeatin (tZ), cis-zeatin (cZ), dihydrozeatin (DHZ), and isopentenyl adenine (iP). These free bases are bioactive forms of cytokinins (Sakakibara 2006; Lomin et al. 2015; Pacifici et al. 2015; Hošek et al. 2020). Cytokines can be classified into two types based on the position of adenine: aromatic CKs and isoprenoid CKs.

Cytokinins coordinate shoot growth with nitrogen fixation in symbiotic root nodules, highlighting their roles in systemic signaling and nutrient uptake (Chen et al. 2022). Cytokinins act as growth promoters by regulating the metabolism and transport of essential nutrients, such as amino acids, carbohydrates, and macronutrients, such as nitrogen, phosphorus, sulfur, and iron (Albrecht and Argueso 2016; Sakakibara 2021) (Fig. 4). Moreover, cytokinins interact with other plant hormones such as auxins and gibberellins to modulate various physiological processes and promote growth (Kokkiligadda et al. 2017). They also play a significant role in stress responses and immunity in plants, as they mediate interactions with salicylic acid in plant immunity, highlighting their involvement in defense mechanisms against pathogens (Argueso et al. 2012). Furthermore, cytokinins have been shown to regulate osmotic stress tolerance, with increased cytokinin signaling leading to growth inhibition and hypersensitivity to osmotic stress (Karunadasa et al. 2020). These findings support the crucial role of cytokinins in balancing growth promotion and plant stress response.

## Role of cytokinins in sweet potato micropropagation

Cytokinins are essential for the micropropagation of sweet potato and influence shoot proliferation, organogenesis, and overall plant tissue growth. Studies have shown that cytokinins such as meta-topolins can enhance shoot production in sweet potato tissue cultures (Amoo et al. 2011). The type and concentration of cytokinins are crucial for successful shoot regeneration in vitro. Several studies have assessed the effects of different cytokinins on sweet potato micropropagation at different concentrations. Most of these studies used shoot tips, nodal segments, and initiated shoots to assess the effects of cytokinins on shoot initiation, regeneration, and multiplication, respectively (Table 1).

**Table 1:** Single use of auxins and cytokinins for in vitro plant regeneration of sweet potatoes using meristematic explants

Genetic material	Medium Supplement	Explant	Results	Reference
UE007	0.3 mg/l BAP	Nodal segment	100% shoot and root formation. 4.21 cm and 2.71 cm for maximum shoot height and root length respectively	Addae-Frim et al. (2014)
Awasa—83	2.0 mg/l BA	Nodal segment	Optimum shoot regeneration (5.26 shoots/explants)	Dugassa and Feyissa (2011)
Awasa local	2.0 mg/l BA	Nodal segment	Optimum shoot regeneration (5.12 shoots/explants)	
Mangawy	2.0 mg/l BA	Nodal segment	Optimum shoot regeneration (5.22 leaves/explant. 2.88 cm shoot length)	Fadaladeen et al. (2022)
Guntute	3.0 mg/l BA	Nodal segment	Optimum shoot regeneration (2.48 shoots/explant)	Dugassa and Feyissa (2011)
Unknown	1.0 mg/l NAA	Nodal segment	Optimum shoot regeneration (86.3% regeneration frequency)	Gong et al. (2005)
Yan Shu–1	2.0 mg/l NAA	Nodal segment	Optimum plantlet development	Ozturk (2021)
Bhu Krishna	2.0 mg/l Meta-Topolin	Nodal segment	Optimum shoot regeneration (20.3 number of shoots with an average length of 5.3 cm	Behera et al. (2024)
Georgia Red	2.5–5.0 mg/l KIN	Nodal segment	Optimum shoot regeneration and rooting	Marco and Walkey, (1992)
Kullufo	0.5 mg/l BA	Lateral bud	Optimum shoot regeneration (19.7 shoots/explant with an average length of 3.97 cm)	Mengs et al. (2018)
White Star	1.0 mg/l BA	Lateral bud	Optimum shoot regeneration (An average of 8.5 shoots/5 weeks)	Litz and Conover (1978)
Kulfo	0.5 mg/l BAP	Shoot tip	Optimum shoot initiation (77.8%) and shoot length (4.4 cm)	Belachew et al. (2020)
	1.0 mg/l BAP	Shoot tip	Optimum shoot multiplication (5.33 shoots/explants)	
Awasa—83	0.1 mg/l IBA	Regenerated shoot	Optimum rooting (6.34 roots/shoot)	Dugassa and Feyissa, (2011)
Kullufo	0.25 mg/l IBA	Regenerated shoot	Optimum rooting (100% root formation)	Mengs et al. (2018)
Kulfo	0.5 mg/l IBA	Regenerated shoot	Optimum rooting (100% root formation)	Belachew et al. (2020)
Bhu Krishna	0.5 mg/l IBA	Regenerated shoot	Optimum rooting (5.8 roots/shoot)	Behera et al. (2024)
Bhu Sona	0.5 mg/l IBA	Regenerated shoot	Maximum rooting	Arathi et al. (2019)
Mabrokat Al-Shimal	1.5 mg/l IBA	Regenerated shoot	Optimum rooting (22.33 roots/explant)	Fadaladeen et al. (2022)
Kullufo	0.5 mg/l NAA	Regenerated shoot	Optimum rooting (96.6% root formation)	Mengs et al. (2018)

Where, *KIN* Kinetin, *NAA* naphthaleneacetic acid, *BA-N6* benzyladenine, *IBA* indole-3-butyric acid, *BAP 6* Benzylaminopurine

Several studies have identified key cytokinin metabolic genes and pathways that regulate shoot development and growth in vitro (Li et al. 2022). This knowledge aids in understanding the genetic and biochemical processes influenced by cytokinins, contributing to improved shoot proliferation and tissue differentiation during sweet potato micropropagation. By leveraging the effects of cytokinins on shoot development, researchers can establish efficient micropropagation protocols for sweet potatoes to generate healthy and genetically uniform plantlets for agricultural and other purposes.

## Interplay between auxins and cytokinins

The interplay between auxins and cytokinins is critical for a wide range of plant developmental processes. It is involved in the mutual control of hormone metabolism, gene expression, and signal transduction pathways (Hussain et al. 2021; Tiwari et al. 2023). It is widely acknowledged that auxins and cytokinins have both synergistic and antagonistic roles (Danilova et al. 2020). Their antagonistic role is exhibited when cytokinins block certain branches of the auxin response pathway, thereby inhibiting specific auxin responses during in vitro organogenesis (Saini et al. 2013). In addition, cytokinins tend to inhibit root elongation, lateral root formation, and hypocotyl growth while reducing shoot chlorophyll content and altering gene expression (To et al. 2008; Zhao et al. 2010; Danova et al. 2018; Tiwari et al. 2023). The synergistic relationship between auxins and cytokinins in sweet potato tissue culture is vital for controlling plant growth and development. Auxins and cytokinins mutually coordinate their activities to control various aspects of plant development including root and shoot growth, organogenesis, and environmental responsiveness (Müller and Sheen 2008; Yang et al. 2017; Mboene Noah et al. 2021; S. Zhao and Li 2021). Crosstalk between auxins and cytokinins occurs at multiple levels and it is essential for the regulation of plant growth and development (Hurný et al. 2020).

The auxin-to-cytokinin ratio is crucial for determining the developmental fate of plant cells in tissue culture. The balance between these two hormones, rather than their absolute levels, plays a significant role in the control of plant development (Danova et al. 2018; Mboene Noah et al. 2021). The appropriate ratio and concentration of auxins and cytokinins in the culture medium significantly influenced the regeneration and growth of the micropropagated sweet potato plants (Table 2). These regulate processes, such as shoot and root formation, callus induction, and organ differentiation in vitro. The coordinated action of these plant growth regulators is crucial for achieving successful outcomes in sweet potato tissue culture.

**Table 2:** Combined use of auxins and cytokinins for in vitro regeneration of sweet potato using meristematic explants

Genetic material	Medium Supplement	Explant	Result	Reference
Kamalasundori	1.5 mg/l BAP + 0.1 mg/l KIN	Nodal segment	Highest percentage shoot initiation (91.3%). Minimum days to shoot initiation (9). Highest number of shoots per explant (11 shoots/explant). Highest shoot length (4.38 cm)	Parvin et al. (2018)
Jewel	0.5 mg/l BAP + 0.5 mg/l KIN + 0.5 mg/l IAA	Nodal segment	Optimum shoot regeneration	Kirakosyan et al. (2022)
Amitchewin	1 mg/l BAP + 0.1 mg/l NAA, 1 mg/l KIN + 0.1 mg/l NAA	Nodal segment	Highest number of initiated shoots (12)	Doussoh et al. (2019)
Abees	2 mg/l BA + oxalic acid 100 mg/l followed by ½MS medium	Nodal segment	Highest shoot number (3.1) and highest node number (16.1)	Dewir et al. (2020)
Purple	3.0 mg/l BAP + 1.0 mg/l NAA	Nodal segment	Optimum shoot multiplication	Arif (2019)
Jewel	0.5 mg/l KIN + 0.5 mg/l IAA	Nodal segment	Highest number of shoots per explant (3.9). highest shoot length (6.1 cm). Highest number of leaves per shoot (9.2)	Kirakosyan et al. (2022)
Jewel	0.5 mg/l BAP + 0.5 mg/l IAA	Regenerated shoot	Highest number of roots per shoot (2.02). Highest number of root length (8.95 cm)	Kirakosyan et al. (2022)
Unknown	0.5 mg/l BA + 0.2 mg/l NAA	Regenerated shoot	Optimum production of rooted shoots (11.47%)	Yang (2010)
Kamalasundori	0.5 mg/l IBA + 0.1 mg/l NAA	Regenerated shoot	Highest percentage shoot initiation (94.12). Minimum number of days for root initiation (6). Highest number of roots per plantlet (9.33). Highest root length (11.13 cm)	Parvin et al. (2018)
Beletech	0.75 mg/l IBA + 0.5 mg/l NAA	Regenerated shoot	Maximum root length (3.4 cm)	Alula et al. (2018)
CN 1108–13	1.0 mg/l BA + 1.0 mg/l IAA	Apical bud	Highest number of plantlets	Kuo et al. (1985)
Beletech	0.75 mg/l BA + 0.5 mg/l KIN	Apical meristem	Minimum days to root induction (3.167 days)	Alula et al. (2018)

Where; *IAA* indole-3-acetic acid, *KIN* Kinetin, *NAA* naphthaleneacetic acid, *BA–N6* benzyladenine, *BAP 6* Benzylaminopurine, mg/l milligram per liter

Moreover, the role of auxins in the regulation of shoot and root growth under osmotic stress conditions has been investigated in sweet potato tissue culture. Auxins, along with other plant growth regulators such as cytokinins, ethylene, and abscisic acid, form a complex hormonal network that affects root growth and development in response to environmental stress (Rowe et al. 2016; Tognetti et al. 2017; Dhar et al. 2020). The interplay between auxins and other PGRs is vital for maintaining a balance between shoot and root growth in micropropagated sweet potatoes. However, the molecular mechanisms underlying these interactions in cultured cells are complex and involve complex regulatory networks. Future experiments should focus on elucidating these mechanisms to aid in proper understanding and scientific applications.

## Factors affecting PGR’s response

Several factors affect the responses of plants to growth regulators. These include genetic factors, type and concentration of growth regulators, plant species, developmental stage, and environmental conditions. Plant genetics significantly influence the response of plants to growth regulators. Variations in gene expression can affect the ability of plants to sense and react to external stimuli, such as nutrient availability, medium composition, light, and temperature (Jemal and Feyissa 2020). Varying responses were recorded among the different sweet potato genotypes subjected to the same concentration of PGR (Table 1). Dewir et al. (2020) investigated the effect of different types and concentrations of cytokinins on axillary shoot multiplication in sweet potatoes. Similarly, Amoo et al. (2011) assessed the effects of different cytokinins on adventitious shoot production from shoot tip explants and recorded varying responses. These experiments suggest that specific types and concentrations of plant growth regulators can also significantly affect plant responses. In addition, different plant genotypes and species may exhibit unique responses to specific growth regulators, owing to differences in their genetic composition. Furthermore, the developmental stage of a plant can influence its sensitivity to growth regulators. Younger plants or tissues may respond differently to mature plants (Jiménez 2005). Optimal environmental conditions, including explant disinfection protocols, are crucial to maximize the effectiveness of growth regulators in promoting in vitro micropropagation (Pérez-Pazos et al. 2023). Plant growth regulators often interact with other hormones and signaling molecules in complex ways. Crosstalk between hormones can also modulate plant responses and influence growth (Malá et al. 2013; Aremu et al. 2014).

## Single and combined effects of auxins and cytokinins

Researchers have extensively investigated the morphological impacts of using auxins and cytokinins, both individually and in combination, on plant regeneration in vitro. Various studies have examined the effects of different types and concentrations of these plant growth regulators on sweet potato in vitro regeneration (Table 1). Dugassa and Feyissa, (2011), and Fadaladeen et al. (2022) conducted similar experiments using nodal segments from different genotypes to evaluate the impact of 2.0 mg/L BA on shoot formation in vitro. Parvin et al. (2018) investigated the combined effects of two cytokinins on shoot regeneration. Their findings showed that nutrient medium supplemented with 1.5 mg/l BAP + 0.1 mg/l KIN produced the highest percentage of shoot initiation (91.3%), shortest time to shoot initiation (9 days), maximum number of shoots per explant (11 shoots/explant), and greatest shoot length (4.38 cm). Studies by Mengs et al. (2018) and Belachew et al. (2020) demonstrated that adding 0.25 mg/L IBA or 0.5 mg/L IBA resulted in 100 percent root formation in regenerated sweet potato shoots from various genotypes. These studies typically used nodal explants for shoot regeneration and regenerated shoots for root formation. Additional research has been conducted to explore the combined use of various auxins and cytokinins at different concentrations for in vitro sweet potato regeneration (Table 2). Kirakosyan et al. (2022) observed optimal shoot regeneration in explants cultured on nutrient media containing 0.5 mg/l BAP + 0.5 mg/l KIN + 0.5 mg/l IAA. It has been found through this study that with different genotypes, the application of 0.5 mg/L to 1 mg/L of BA or BAP tends to yield optimum shoot multiplication with 0.5 mg/L of IBA yielding optimum rooting. Therefore, the adoption of these concentrations for plant regeneration could be significantly beneficial to plant producers and researchers.

## Conclusions and future prospects

In conclusion, this comprehensive review highlights the significant role of plant growth regulators, particularly auxins and cytokinins, in sweet potato micropropagation. The study reveals that 6-benzylaminopurine (BAP) and N6 benzyladenine (BA) are the most frequently used synthetic cytokinins, while indole-3-butyric acid (IBA) and naphthaleneacetic acid (NAA) are the preferred synthetic auxins. Optimal concentrations for shoot multiplication and rooting have been identified, providing valuable insights for plant producers and researchers. However, this review also uncovers a critical gap in the current research landscape: the limited focus on the influence of auxins and cytokinins on sweet potato in vitro rooting. Given the increasing challenges posed by climate change and its impact on crop production, there is an urgent need to explore innovative methods to enhance crop resilience. The development of an extensive root system, crucial for plant survival under abiotic stresses and higher yield in sweet potatoes, emerges as a promising area for future research. This study sets the stage for further investigations into the effects of auxins and cytokinins on sweet potato rooting, as well as their impact on the physiological development and abiotic stress responses of sweet potatoes. Such research has the potential to contribute significantly to food security and sustainable agriculture in the face of changing environmental conditions. By addressing these knowledge gaps, future studies can pave the way for more resilient and productive sweet potato varieties, ultimately benefiting millions of people who rely on this important staple crop worldwide.

## Data Availability

Not Applicable.
